# Cerebral rheumatoid vasculitis: a case report

**DOI:** 10.1186/1752-1947-6-302

**Published:** 2012-09-13

**Authors:** Rim Akrout, Samar Bendjemaa, Hela Fourati, Mariem Ezzeddine, Imène Hachicha, Chokri Mhiri, Soufiene Baklouti

**Affiliations:** 1 Rheumatology Department, Hedi Chaker Hospital, Sfax, Tunisia; 2 Neurology Department, Habib Bourguiba Hospital, Sfax, Tunisia

**Keywords:** Cerebral vasculitis, Rheumatoid arthritis, Cerebral magnetic resonance imaging, Central nervous system

## Abstract

**Introduction:**

Central nervous system involvement in rheumatoid arthritis is infrequent. The most frequent neurological manifestations of rheumatoid arthritis are peripheral neuropathy and cervical spinal cord compression due to subluxation of the cervical vertebrae. Cerebral rheumatoid vasculitis is an uncommon and serious complication which can be life-threatening.

**Case presentation:**

A 52-year-old North African Tunisian Caucasian woman presented with a six-week history of headache. She had suffered seropositive and destructive rheumatoid arthritis for nine years without any extra-articular complications. Magnetic resonance imaging of the brain with the T2 sequence showed high-intensity signal images at the frontal and parietal cortico-subcortical junction suggesting hemispheric vasculitis.

**Conclusions:**

Cerebral vasculitis is an infrequent complication in rheumatoid arthritis which is associated with high morbidity and in some cases can be life-threatening. Early assessment and a high index of suspicion to recognize such complications are essential in managing these patients.

## Introduction

Vasculitis is a group of chronic inflammatory diseases in which the blood vessel is the target of an immune reaction. They can be idiopathic or due to infection, neoplasm, collagenoses or drugs. Rheumatoid arthritis (RA) is a chronic, progressive, systemic inflammatory disorder in which the joints are the primary target. Inflammatory central nervous system lesions are infrequent in RA. Cerebral rheumatoid vasculitis is an uncommon and serious complication of RA. Most reported cases have led to the death of the patient especially when cerebral vasculitis was associated with systemic rheumatoid vasculitis (Table 1). The treatment of such patients must be effective. There is no standard treatment of this rare complication. We describe a patient with longstanding RA and isolated central nervous system vasculitis.

**Table 1:** Literature review

**Reference**	**Age / Sex**	**Years**	**Pathologic diagnosis**	**Neurological manifestation**	**Treatement**	**Outcome**
Pirani and Bennet, 1951 [1]	22 Male	16	CP and systemic vasculitis	Depressed MS, seizures	NR	Exitus
Sokoloff and Bunim, 1957 [2]	64 Male	30	CP and systemic vasculitis	NR	GC	Exitus
Johnson *et al*. 1959 [3]	37 Female	1 year 8 month	CP and systemic vasculitis	Seizures	GC	Exitus
Johnson *et al.* 1959 [3]	63 Male	3	CP and systemic vasculitis	Hemiparesis	GC	Exitus
Steiner and Gellbloom, 1959 [4]	62 Male	20	CP vasculitis	CN dysfunction, depressed MS	GC	Exitus
Ouyang *et al*. 1967 [5]	58 Female	30	CP vasculitis	Seizures, hemiparesis	GC	Exitus
Ramos and Mandybur, 1975 [6]	63 Male	1	CP and systemic vasculitis	Gerstmann syndrome	NR	Exitus
Watson *et al*. 1977 [7]	54 Female	20	CP vasculitis	CN dysfunction, aphasia, hemiparesis, ataxia	GC	Exitus
Kiss *et al.* 2006 [8]	51 Female	39	CP and systemic vasculitis	Hemiparesis	GC immunoglobulin	Exitus
Rodriguez *et al*. 2006 [9]	49 Female	10	CP vascultis	Aphasia, hemianopia	GC cyclophosphamide	Improvement
Rodriguez *et al*. 2006 [9]	70 Female	Recent diagnosis	CP vascultis	Seizures	GC cyclophosphamide	Improvement
Mrabet *et al.* 2007 [10]	59 Female	20	CP vascultis	Headache, diplopia, and gait disorders	High-dose GC cyclophosphamide	Improvement
Caballol Pons *et al*. 2010 [11]	71 Female	15	CP vascultis	Headache, dysarthria	High-dose GC	Improvement
Ohno *et al*. 1994 [12]	46 Female	16	CP vasculitis	dysarthria and left hemiparesis	methotrexate therapy	Improvement
Present case	52 Female	9	CP vascultis	Headache	Intensification MTX therapy	Improvement

CN, cranial nerve; CP, cerebral parenchymal; GC, glucocorticoid; MTX, methotrexate; MS, mental status; NR, not reported.

## Case presentation

**Figure 1:**
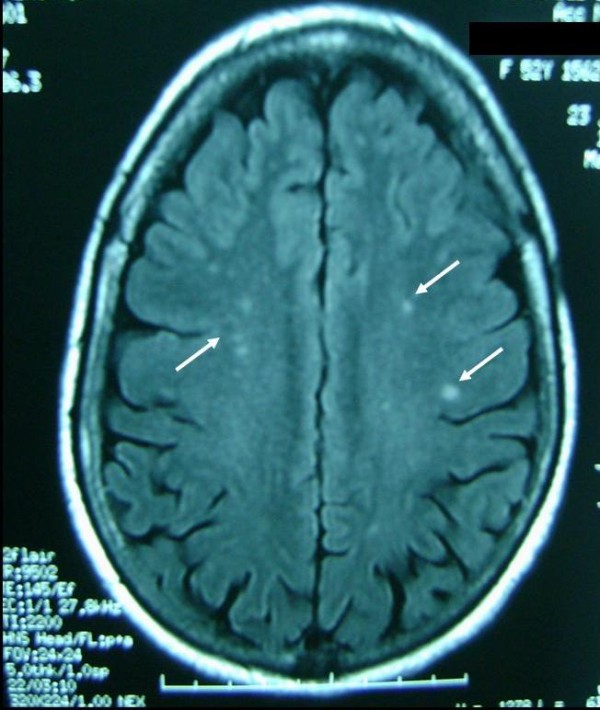
**Cerebral magnetic resonance imaging.** T2-weighted image demonstrates high-intensity-signal involving white matter of two cerebral hemispheres.

**Figure 2:**
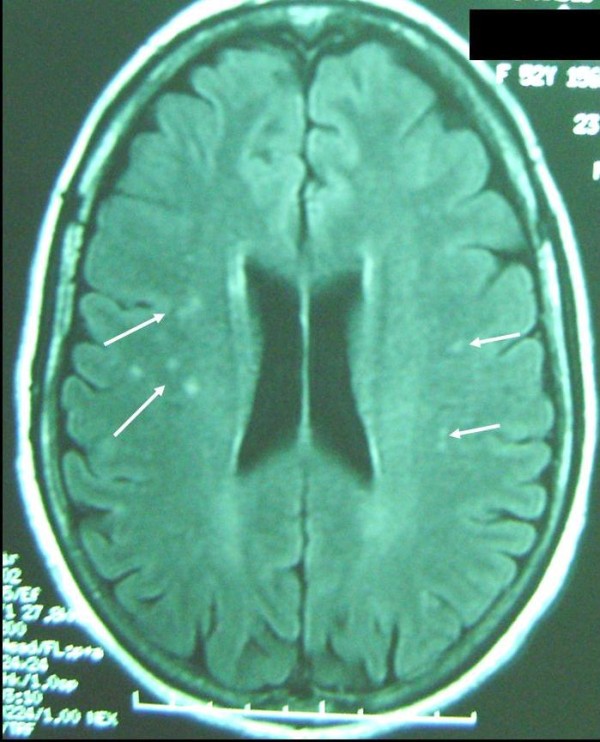
**Cerebral magnetic resonance imaging.** T2 sequence, axial section demonstrates high signal in the subcortical area of the frontal and parietal lobes.

A 52-year-old North African Tunisian Caucasian woman was admitted with a six-week history of headache to hospital. She had been diagnosed with seropositive destructive RA nine years before, without any extra-articular manifestations. She received methotrexate 7.5mg per week, low doses of prednisone 7.5mg per day and anti-inflammatory drugs. She presented with a six-week history of bilateral temporal headaches. There was no fever or vomiting. Her body temperature was normal and her blood pressure was 120/60mmHg. Physical examination revealed typical joint deformities of RA, but with no subcutaneous nodules or skin lesions. Synovitis was noted at both wrists as well as at the second and third metacarpophalangeal joints of both hands. Her neurological examination was normal including her deep tendon and plantar reflexes. There was no evidence of meningitis or focal neurological signs. Her temporal pulses were brisk and symmetric. Laboratory tests revealed the following results: erythrocyte sedimentation rate of 27mm/hour, C-reactive protein of 14mg/l (normal: <6) and hemoglobin of 14.9mg/DL. The white blood cell count was normal (8180/mm3) as were her platelets (380,000/mm3). Liver and kidney function tests were normal, as well as blood glucose levels. No obvious infection, disseminated intravascular coagulation, atlanto-axial dislocation or other collagen diseases were recognized by physical and blood examinations. Electrophysiological studies showed no evidence of peripheral nerve lesion or denervation. Tests were positive for rheumatoid factor (512UI/ml) and for antinuclear antibodies (1/640). Her serum complement, circulating immune complexes, and antineutrophil cytoplasm antibodies were normal. The fundus examination and the fluorescein angiography did not reveal any signs of vasculitis. Magnetic resonance imaging (MRI) of her brain with the T2 sequence showed high-intensity signal images at the frontal and parietal-cortico-subcortical junction suggesting hemispheric vasculitis (Figure 1, 2). Our patient had not had any severe neurological manifestation or any systemic non-neurological manifestation. She suffered only from persistent headache. Intensification of the methotrexate therapy (15mg per week) was enough to give a good outcome. Her headache disappeared in two weeks and there were no symptoms of meningitis or focal neurological signs after four months.

## Discussion

It is well documented that collagen diseases, such as systemic lupus erythematosis (SLE), Sjögren’s syndrome, and Behçet’s disease, are often complicated by cerebrovascular disorders [13]; however, these seldom occur in RA [14]. The rate of occurrence of cerebral vasculitis in patients with RA is 1% to 8% [15]. Neurological manifestations in patients with RA can be due to inflammatory central nervous system lesions. They have been described traditionally in seropositive patients with long-standing, active and erosive RA, with subcutaneous nodules and extra-articular manifestations [11]. In our case, the RA was seropositive, active and erosive but the patient had no extra-articular signs especially no subcutaneous nodules. Cerebral vasculitis is usually associated with severe general signs as well as prominent extra-articular manifestations with minimal joint manifestations [10].

Our patient had no extra-articular manifestations other than those related to cerebral vasculitis. Neurological involvement in RA includes atlantoaxial subluxation, polymyositis, mononeuritis multiplex, peripheral neuropathy, rheumatoid nodules in the central or peripheral nervous system, and rheumatoid vasculitis causing stroke and/or neuropathy [13]. The neurological manifestations of rheumatoid cerebral vasculitis include focal signs such as hemiplegia, partial epilepsy, cranial nerve involvement, or visual field loss, altered consciousness, confusion, and cognitive impairment or dementia [10]. In our case, vasculitis is revealed by severe and persistent headache which is a common symptom and may suggest giant cell arteritis, particularly as this condition can occur in patients with RA [16]. The presence of temporal pulse and the normality of the funduscopy, especially the absence of vasculitis signs, militated against giant cell arteritis. Patients with a diagnosis of pathological vasculitis involving the cerebral parenchyma are infrequent [1-9]. Eight specified cases were considered to show isolated cerebral vasculitis, and six other cases were associated with symptoms indicating systemic rheumatoid vasculitis. The present case report is similar to the majority of previously reported cases in that our patient displayed an isolated central nervous system vasculitis. We did not detect any other visceral vasculitis. Our patient had a long-standing history of RA, which required steroid therapy and the diagnosis of rheumatoid cerebral vasculitis was made by cerebral MRI. Other causes of cerebral vasculitis were eliminated as far as possible based on past history, physical findings, clinical data, laboratory studies, and response to the therapy.

Biopsy of the brain is not systematic. Actually, neuroradiological analysis can be useful for detecting cerebral vascular disorders. In our case, T2-weighted MRI showed high signal intensity in the frontal and parietal cortico-subcortical junction, however, no obvious abnormality was detected on T1-weighted MRI. Glucocorticoids at different dosages and administrations have been reported in the treatment of central nervous system rheumatoid vasculitis. Several alternatives such as azathioprine [17], intravenous immunoglobulins [8] and cyclophosphamide [9,18] are available for patients with corticosteroid-resistant or refractory vasculitis. In our case, the intensification of the methotrexate therapy was enough to give a good outcome, especially as there were no symptoms of systemic vasculitis. Tatsuharu Ohno [12] reported the successful management of cerebral vasculitis in a 46-year-old woman who presented with sudden dysarthria and left hemiparesis after the initiation of the methotrexate therapy [12]. Although high doses of glucocorticoid and cyclophosphamide were used, the cases associated with systemic rheumatoid vasculitis had the worst prognosis.

## Conclusions

Neurological involvement in rheumatic disease is associated with high morbidity and, in some cases, can be life-threatening. Early assessment and a high index of suspicion for recognized complications are essential in managing such patients. Although serious neurological complications in rheumatic disease appear to be rare, few studies have been conducted on their prevalence. Studies of larger cohorts of patients in multi-center settings are required to assess the management of such patients more fully.

## Consent

Written informed consent was obtained from the patient for publication of this case report and any accompanying images. A copy of the written consent is available for review by the Editor-in-Chief of this journal.

## Competing interests

The authors declare that they have no competing interests.

## Authors’ contributions

All the authors of this article participated in the clinical work-up, the medical photography, the literature search and the writing of the manuscript. All authors read and approved the final manuscript.
